# ‘You can't stay away from your family’: a qualitative study of the ongoing ties and future plans of South African health workers in the United Kingdom

**DOI:** 10.3402/gha.v8.26125

**Published:** 2015-03-17

**Authors:** Katherine Taylor, Claire Blacklock, Gail Hayward, Posy Bidwell, Pallavi Laxmikanth, Nicholas Riches, Merlin Willcox, Shabir Moosa, David Mant

**Affiliations:** 1 Nuffield Department of Primary Care Health Sciences, Oxford University, Oxford, UK; 2 Centre for Primary Care, University of Manchester, Manchester, UK; 3 Department of Family Medicine, Faculty of Health Sciences, University of Witwatersrand, Johannesburg, South Africa

**Keywords:** migration, South Africa, United Kingdom, doctors, nurses

## Abstract

**Background:**

Migration of African-trained health workers to countries with higher health care worker densities adds to the severe shortage of health personnel in many African countries. Policy initiatives to reduce migration levels are informed by many studies exploring the reasons for the original decision to migrate. In contrast, there is little evidence to inform policies designed to facilitate health workers returning home or providing other forms of support to the health system of their home country.

**Objective:**

This study explores the links that South African-trained health workers who now live and work in the United Kingdom maintain with their country of training and what their future migration plans may be.

**Design:**

Semi-structured interviews were conducted with South African trained health workers who are now living in the United Kingdom. Data extracts from the interviews relating to current links with South Africa and future migration plans were studied.

**Results:**

All 16 participants reported strong ongoing ties with South Africa, particularly through active communication with family and friends, both face-to-face and remotely. Being South African was a significant part of their personal identity, and many made frequent visits to South Africa. These visits sometimes incorporated professional activities such as medical work, teaching, and charitable or business ventures in South Africa. The presence and location of family and spouse were of principal importance in helping South African-trained health care workers decide whether to return permanently to work in South Africa. Professional aspirations and sense of duty were also important motivators to both returning and to being involved in initiatives remotely from the United Kingdom.

**Conclusions:**

The main barrier to returning home was usually the development of stronger family ties in the United Kingdom than in South Africa. The issues that prompted the original migration decision, such as security and education, also remained important reasons to remain in the United Kingdom as long as they were perceived as unresolved at home. However, the strong residual feeling of identity and regular ongoing communication meant that most participants expressed a sense of duty to their home country, even if they were unlikely to return to live there full-time. This is a resource for training and short-term support that could be utilised to the benefit of African health care systems.

The health workforce as one of the core building blocks of a health system needs to be sufficient in number, skill mix, and distribution (WHO, The WHO Health Systems Framework). Studies have shown a significant positive correlation between low health worker density and under-five mortality (1), as well as non-achievement of specific goals such as coverage of measles immunisations (2). Physician density in sub-Saharan Africa ranges from 0.08 per 1,000 population in Tanzania to 0.77 per 1,000 population in South Africa, in contrast to 2.79 per 1,000 in the United Kingdom in 2012 (3). Similarly, nursing and midwife personnel density was 0.14 per 1,000 population in Niger, 4.7 in South Africa and 8.8 in the United Kingdom (4). The health worker inequity crisis is fuelled by insufficient training capacity in many countries in addition to emigration to countries with higher health worker densities, despite codes of practice developed by the WHO (5) and by individual countries such as the United Kingdom (6). Little is known about links that migrant health workers maintain with their home countries post-migration or their future migration plans, which could influence policies to encourage their return.

Historically, a significant proportion of South African-trained health workers have emigrated post-training but these numbers have increased in recent decades. For example when surveyed in 1975, 20% of medical graduates who had qualified between 1925 and 1972 from the University of Witswatersrand in South Africa were located abroad (7), but by 1998 this proportion had more than doubled to 45% (8). Popular destinations are Australia, Canada, the United States, and the United Kingdom (9). In the United Kingdom, where 36% of doctors on the current List of Registered Practitioners qualified outside the United Kingdom, South Africa is the third most frequent country of training, after India and Pakistan (10). South African Department of Health statistics for 2006 show that 7,000 South African nurses were working abroad, compared to a nursing workforce in South Africa of 66,000 that year (11). Data using destination country census information found the number of South African nurses working in nine receiving countries was equivalent to 5% of the South African nursing workforce in 2000 (12).

The reasons for health worker migration from Africa, and more specifically South Africa, have already been studied (13). There are several factors that make the United Kingdom an obvious destination for emigrating South African-trained health workers. These factors include non-professional links such as historical colonial ties, ease of travel between the two countries with little time difference and direct flights, large expatriate communities, frequency of dual citizenship, common language, as well as professional factors such as previous active recruitment (14) and similar medical school academic standards. A meta-analysis of qualitative studies looking at this issue across Africa found six major themes leading to migration, including financial pressures, strain within the workplace, and personal or family security (13). Similarly, in South Africa financial factors (15), insecurity, crime, and racial tension (16) were the main reasons for emigration. There has been less emphasis on ongoing links that migrant health workers keep with their country of training, the potential benefits of these links, and how they might influence migratory intent, particularly return migration. Crush and Williams (17) exemplify a network of South African professionals abroad and how this network encourages collaboration with South African counterparts.

Regarding future career plans of migrant health workers, Kangasniemi et al. studied phone and postal questionnaires of migrant doctors working in the United Kingdom and found that 50% of those from low-income countries did intend to return to their home country (18). The results of a postal survey of international nurses working in London found that South African nurses were more likely to plan to stay for 2–5 years, whereas nurses from other African countries were most likely to plan to stay for over 5 years (19). Although these studies provide a useful picture of future migration intentions, there is currently no information about the ongoing ties that migrant health workers have with their countries of origin, nor qualitative studies regarding their future plans. This paper presents the links that South African-trained health workers working in the United Kingdom maintain with their country of training, in a detailed qualitative study. Any ongoing links may have a positive or negative effect on their views of South Africa and decisions about future career plans, which could be important for workforce planning and building on relationships and pull factors back to work in South Africa.

## Aims of this study

1. To explore the links that South African-trained health workers who now live and work in the United Kingdom maintain with their country of training.
2. To explore the future migration plans of interviewees and the factors influencing those decisions.

## Methods

The study aimed to capture the experiences of individuals living in the United Kingdom who had trained as a doctor, nurse, or midwife in South Africa. A purposive sample was recruited to include individuals of different ethnicity, gender, age, professional training, length of stay in the United Kingdom, parental status, and current role in the United Kingdom. We contacted national professional organisations and support groups, local health employers, and individuals known to the research team in order to recruit participants. Posters were displayed and adverts were sent to targeted groups and individuals. People expressing an interest were sent a study information pack that included an information sheet, a response form, and a consent form (20).

**Fig. 1:**
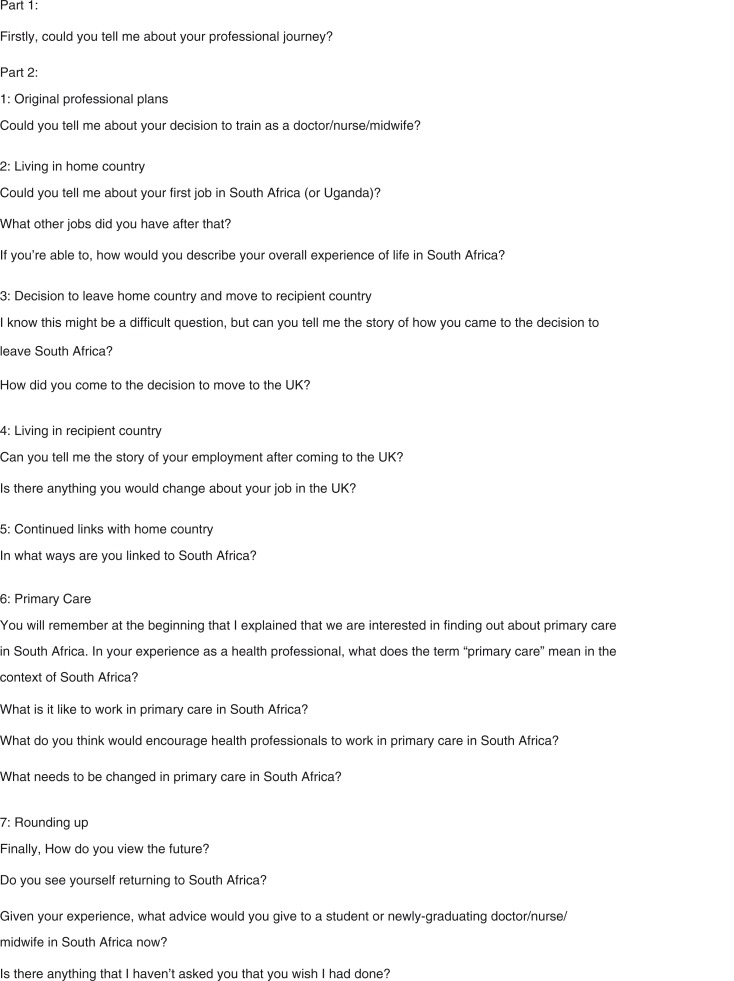
Example initial interview schedule.

Interviews took place between January and August 2012. Semi-structured interviews lasting around 60 min were conducted by experienced qualitative researchers (CB, GH, NR, or PL), in a place of the participant's choice, usually their home or office. A topic guide was used that had been constructed from existing literature and was adapted as themes emerged from the interviews. After an introduction to the background of the study, participants were asked to describe their professional journey, starting from their decision to train as a health care professional. Topics covered during the interview included original migration decision making, experiences of working in the United Kingdom, views of primary care in South Africa, ongoing links with South Africa, and future plans (see Fig. 1). The interview schedule was modified during data collection, enabling researchers to accommodate emergent themes; interviews were conducted until thematic saturation was reached. Interviews were audio recorded, transcribed verbatim, and anonymised.

During the analysis transcripts were read and reread, and sections of the transcripts that related to 1) current links with South Africa and 2) future intentions were independently coded using NVivo 10 (qualitative data management software) by two researchers (KT, CB). Any disagreements regarding selected text and creation of codes were discussed and resolved. The data extracts for each code were examined to identify the main themes within each of these two areas of interest. These extracts were considered in relation to what else was known about each participant (profession, intention to stay in United Kingdom or return, whether they have children, etc.), grouped under the headings that are presented in the paper, and illustrated with extracts from the transcripts. Both researchers were UK-trained GPs – one had previously worked in South Africa (KT) and the other elsewhere in Africa (CB).

The study was approved by the Research Ethics Committee of the University of Oxford (ref: MSD/IDREC/C1/2011/96) and formed part of the HURAPRIM study (http://www.huraprim.ugent.be/drupal/).

## Results

We interviewed 6 South African-trained doctors and 10 nurses (of whom five were also trained midwives). Nine participants were working in the same professional role in the United Kingdom as in South Africa, and seven were working in different roles. Despite purposive sampling, the majority of participants were female, white and over 35 years of age. Half of the participants had children.

### Ongoing links with South Africa

Major themes identified were family, friends, and personal identity; active communication and comparison; and charitable and professional links.

#### Family, friends, and personal identity

All participants had family members or friends still living in South Africa. These ties were very important for many participants and were a difficult aspect of living in the United Kingdom.

… it is sometimes hard to be far away. You know my parents still live in South Africa and if they fall ill, they're quite elderly, if they – you know – something's the matter with them, it's hard because you're far away. You can't just quickly go for a cup of tea …. (Male doctor, participant 13 – ‘p13’)

In addition, many participants talked about their own personal identity as a lasting tie with South Africa.

I am still a South African. I, everything, my whole family is in South Africa still. (Female nurse, p1)

#### Active communication and comparison

Advances in information technology such as online social networks and internet-based international calling were important tools of communication, in addition to phone calls and face-to-face reunions. Active channels of communication with family, friends, and peers from South Africa kept interviewees informed about what others were now doing, and many used this to make comparisons with their own situation.

We Skype a lot, we phone a lot, we're texting all the time and I still phone friends over there who are still working in the clinics … I left. And I still compare notes, what is happening in this side of life? Are your people doing this? Are your people doing that? So I'm still comparing but not as much as I was when I first came over. (Female nurse midwife, p8)

… my colleagues have moved far, they are liberated mentally and are on the senior positions where they do policies … they are able to shape the health system in South Africa. (Female nurse midwife, p14)

Comparisons also extended to those who had migrated from South Africa, to countries other than the United Kingdom.

… and now the information's coming back for the last five, ten years so strongly of how great life is in Canada compared to in South Africa and the ones who've been to the States or Australia, the more the information comes in the more people are leaving and so when you have these reunions … they'll convince two or three other people and I see on Facebook a year later those two or three people I was talking to had just spoken to the other friends are on their way to Canada and Australia and that's it. (Male doctor, p12)

In addition, communication allowed discussion and insights into the wider political and health system environment in South Africa that they had left and whether any changes were occurring.

I spoke to my mum just the other day and it seems like they are upgrading the hospitals and things like that. Staffing-wise, I don't know if it'll ever get better. (Female nurse, p5)

Almost all respondents reported making frequent visits to South Africa from the United Kingdom, the main purpose being to see family and friends. In addition to maintaining important relationships, visits gave insight into the political and social climate, allowing comparisons to be made between their current life in the United Kingdom and that in South Africa prior to emigration.

Now when I go back it's different. Now I can sort of see it with a, I see it with a different view. I'm still very aware and I'm still very much living with alarms and all the rest of it and I live like that here, still, but it's not quite as bad. (Female nurse midwife, p4)

#### Charitable and professional links

Some respondents also engaged in other activities, such as charitable ventures (e.g. setting up scholarship programmes, volunteer work, or teaching).

Those times I used to go to South Africa and when I was on holiday I would be asked in my hospital during my, you know, my holiday to go there and talk about you know, nursing care or … how to look after children or immunisation or that kind of thing … (Female nurse midwife, p6)

Then in South Africa, in my rural area [I] decided to set up this NGO which is offering scholarships to the orphans because of that. (Female nurse, p10)

A variety of professional links were reported, ranging from clinical work, business projects, involvement in diaspora networks and research.

… at the moment I've got two start-up businesses in South Africa in health care that are running which I started during the MBA …. (Male doctor, p12)

There were two interviewees who had retained their professional registration in South Africa while working in the United Kingdom, one for the purpose of working in South Africa during holidays, and the other as their registration had not yet expired. There were however some interviewees who had no professional links at all.

### Future plans

The future plans of respondents were split between those who intended to stay in the United Kingdom and those who planned to return to South Africa, as well a couple who were still undecided upon their future career location.

I feel that I can contribute more there [South Africa] than I can here, I feel that I've learned skills here that I can take back. (Male doctor, p15, about returning to South Africa)

It will always be a nice place for us to go and visit [South Africa] but we're very happy living here. (Male doctor, p13, about living in the United Kingdom)

Major themes identified were the following: family factors; a sense of duty to South Africa; social factors; and professional factors.

#### Family factors

Family factors were often the principal factor influencing the decision as to whether to return to South Africa or not.

I mean you can't stay away from your family so I'm going to join my family. (Female nurse midwife, p14, about returning to South Africa)

We're very happy here [the UK] as a family and we made the decision at the time and decided it would probably be a very long-term decision and felt the need to become part of the community and integrate … it truly feels like home to us now and my children having grown up here and this truly I think is home for them. (Male doctor, p13, about staying in the United Kingdom)

For many who planned to remain in the United Kingdom, their children had established themselves in the UK education system, and some were now taking important exams or preparing to go to university. Spouses also played an important role in decision making; the decision to remain was therefore taken as a family unit.

#### Sense of duty to South Africa

For those respondents planning to return to South Africa, many articulated a strong sense of purpose and responsibility that influenced their decision making.

… I could never stay here and work in the NHS and pretend that I don't know what I do know, because I've seen what I've seen and I can't ignore it and I know that I could live a very comfortable life in the UK but I would never be happy … yah. (Female doctor, p16)

A strong sense of duty to South Africa was not exclusive however to those intending to return; one nurse who intended to remain in the United Kingdom made the following statement about her charitable work:

I'll never stop doing the job I'm doing in South Africa because when you look at the contribution of other people from different countries towards development of South Africa you ask yourself, ‘Why am I not doing anything to help my community?’ (Female nurse, p9)

#### Social factors

Some of those intending to return spoke about the better quality of life in South Africa.

… It's about quality of life as well. I think we have a better quality of life there [South Africa] even though there's crime yes …. (Female nurse, p5, intending to return)

A couple of respondents however discussed negative aspects of the wider social environment, including financial security, crime, and the political environment, that were influencing their decision as to whether to return.

I'm very happy to be in South Africa, very happy to work, very happy to build things and make; but to be honest I'm only happy as long as I can get out the country whenever I want and know that I could work in the UK or somewhere else and that my asset base will be safe and I would not want to raise a family in South Africa …. (Male doctor, p12, returning temporarily)

Some interviewees had plans to move back temporarily, or were still undecided about their future destination.

South Africa's always home so maybe in ten years’ time maybe yes I will go back there. I'd love to be able to live in both, I'd love to spend my … UK winters in Cape Town and UK summers be here. (Female doctor, p7, undecided)

#### Professional factors

Many mentioned work-related factors affecting their future intentions, and there was a wide range of planned career pathways including clinical, teaching, health policy, research, and private enterprise. There was recognition of the differences between the working environment in South Africa and the United Kingdom; some found aspects of the work environment in South Africa preferable:

… just purely … from a medical point of view I find that work more interesting. I find the work here a bit boring. (Male doctor, p15)

I think in South Africa there's much more respect for the profession which I don't find in the UK. And that would be one of the reasons why we'd go back. (Female nurse, p5)

However, some saw the differences as a barrier to continuing their previous professional role if they returned to South Africa.

At times I do consider returning and I find myself getting put off from returning because of the challenge it would bring in terms of practical skills. I've lost so many skills from a practical perspective since I've left and also the theoretical knowledge around the types of conditions you see. What you see in day to day practising in England is very, very different to what you see in day to day practice in rural Africa. (Male doctor, p10)

Others discussed the practical difficulties relating to employment opportunities as barriers in South Africa.

… that would worry me to think that you know, I have opportunities here [the UK] which I might give up and then I'm unemployable when I get back, so that's a big concern to me although I haven't actually you know, put my foot in the water and say ‘I'm here’ sort of thing. But that does worry me because before I left I could see that all of the posts were all going and they were looking at, you know, the languages, etc., you know, you would need to, you would need to at least hold one African language, which I don't really, so that would be quite difficult for me. (Female nurse midwife, p4)

## Discussion

This paper presents a detailed study of the ongoing ties that South African-trained health workers who have migrated to the United Kingdom have with South Africa, alongside their future career migration plans and the factors that influence these decisions. A major finding was the ongoing communication between the health workers and their family, friends, and colleagues in South Africa and across the world via social media, telephone, diaspora networks, and reunions. This was alongside frequent visits to their ‘home country,’ giving the South African diaspora an up-to-date view on crime and safety, but also health care facilities, job opportunities, and standard of living at home and also in other destination countries. The study has revealed a wide variety of professional and charitable links that the South African diaspora can have with their country of training, regardless of whether they intend to move back there.

The issue of crime and safety for professionals at work and their families is a persistent problem that has been shown to be a major reason for health workers to emigrate from South Africa (16). Despite these social factors and perceived professional barriers, we found that when discussing migrating on from the United Kingdom the respondents mainly intended to return to South Africa. This finding contrasts with the Humphries study (21), in which participants intending to leave their destination country didn't intend to return to their country of origin. For those participants not intending to return to South Africa, their new family in the United Kingdom was the main ‘stay’ factor, as explored in Padarath's framework of ‘stick’ and ‘stay’ factors (22). This family influence implies that interventions and initiatives to encourage migrants to return to work in South Africa have a limited potential impact once health workers and their families have become settled in the United Kingdom. To obtain the educational experiences that are sought (23) while avoiding roots being made in the United Kingdom, future research could look at whether it is beneficial to promote shorter-term exchanges during or soon after training.

As well as looking at ways to reduce migration or encourage migrants to return, the strong feelings of responsibility that migrated South African health workers have to their ‘community’ also indicates a potential resource that could be harnessed. Alongside focusing on the reasons that professionals emigrate, easing the professional transition back to their home country may be helpful; although there are a number of organisations that link professionals to work opportunities, they do not directly address the upskilling or adaptation required for a different clinical environment. Opportunities for those intending to stay in the United Kingdom but wanting to have an ongoing professional or charitable input in South Africa might include short-term projects, teaching, or mentoring programmes such as through charitable organisations (24).

When looking at policies to encourage medical diaspora to return professionally to South Africa, they will have limited impact when family is the decisive factor, therefore this study adds weight to the argument that the original motivation to leave South Africa and career plans at that stage have a lasting impact.

### Strengths of the study

This is the first qualitative study exploring the future plans and ongoing ties to South Africa of South African-trained health professionals who have migrated to the United Kingdom. Qualitative data collection and analysis allowed depth and breadth of experience to be explored.

### Limitations of the study

Although this is a small study, we believe that data saturation was reached on the two main areas of interest because the last few interviews did not add new perspectives. Future work could try to include more non-white and male professionals, who were under-represented in this sample. This study was able to access a broad set of opinions, but we did not collect some socio-demographic and life-course data that we think could have helped to examine patterns in the responses, e.g. duration in the United Kingdom, years prior to retirement, whether parents are living in South Africa, and ages of children. We recommend that future work should include more detail on these variables.

## Conclusions

After migration to the United Kingdom, South African health workers maintain strong links with their country of training, through family and friends, and often professional links as well. Many express a strong sense of responsibility to South Africa. Once a health worker has left South Africa and is living in the United Kingdom, their family in the United Kingdom is one of the most significant reasons preventing their return. Finally, it is worth acknowledging that, although many of those who do emigrate do not intend to return, the majority still see South Africa as their identity, or ‘home,’ and wish to repay something to their community. Further research is needed to find the most effective ways of building on these motivations for the benefit of South Africa.
